# Prevalence of dog-mediated rabies in Ethiopia: a systematic review and Meta-analysis from 2010 to 2020

**DOI:** 10.1186/s42522-021-00046-7

**Published:** 2021-08-04

**Authors:** Shiret Belete, Melke Meseret, Haileyesus Dejene, Ayalew Assefa

**Affiliations:** 1 Department of Veterinary Medicine, Woldia University, Woldia, Ethiopia; 2 College of Veterinary Medicine, University of Gondar, Gondar, Ethiopia

**Keywords:** Dog bite, Ethiopia, Meta-analysis, Rabies

## Abstract

**Background:**

Ethiopia accommodates the second largest number of human rabies deaths in Africa. This systematic review and meta-analysis aimed to summarize and pool estimates of dog-mediated rabies status in Ethiopia.

**Methods:**

Published researches between 2010 and 2020 were comprehensively searched and the required information was extracted. The prevalence was estimated using the random-effects meta-analysis because higher heterogeneity between studies was expected.

**Results:**

The pooled estimate of rabies was 32% (95% CI: 19–46%), with individual study prevalence estimates ranged from 1 to 78%. Studies were approximately weighted equally with individual weight ranging from 5.19–5.28%. Subgroup analysis indicated that the random pooled prevalence of rabies was 28% (95% CI: 0–81%) in animals and 33% (95% CI: 20–47%) in humans. Furthermore, a subgroup analysis across regions indicated that the pooled prevalence was 78% in Addis Ababa, 46% in Oromia, 40% in Tigray and 5% in Amhara regional states. No single study was reported from the country’s eastern and southern parts to be included in this meta-analysis.

**Conclusion:**

The estimated pooled rabies prevalence was found high and showed varying among study regions. Therefore, focusing on mass dog vaccination campaigns and public awareness should be implemented to control the disease.

**Supplementary Information:**

The online version contains supplementary material available at 10.1186/s42522-021-00046-7.

## Introduction

Rabies is a life-threatening, zoonotic viral disease that can cause fatal encephalomyelitis [12]. According to the WHO [19] canine rabies causes an estimated 61,000 deaths per year within the wider international community, of which 56 and 44% of the deaths occurred in Asia and Africa, respectively [6]. This disease is mainly transmitted by dog-bite and causes significant morbidity and mortality among humans and animals with high incidence in rural areas each year [19].

The virus has long been a significant public health threat in Ethiopia [5].

Yimer et al. [21] and Deressa et al. [5] reported dogs are the primary cause of fatal human rabies and responsible for maintaining and disseminating rabies in Ethiopia. The country accommodates the second largest number of rabies deaths of all African countries [4]*.* The first rabies epidemic in Ethiopia was recorded in Addis Ababa in 1903 [15]. A retrospective study done between 2001 and 2009 by the Ethiopian Public Health Institute (EPHI) showed that approximately 1000 to1600 patients were exposed each year in Addis Ababa [5, 17]. The total number of animal rabies cases in Ethiopia remains unknown; however, with a rural and farming population of more than 80%, annual livestock losses caused by rabies pose a significant societal and economic burden [16].

In recent data reported by Beyene et al. [3], more than 2900 human rabies deaths occurred every year. The annual rabid dog exposures in some selected urban and rural districts were estimated at 135,101 and 86 bites per 100,000 inhabitants, respectively [2].

Dog-derived rabies in rural seating has also been reported as a potential problem for animal production sector. In most rural parts of the country, dogs are kept in close contact with other livestock for safeguarding purposes, which might provide an opportunity to transmit and maintain the virus in the population [12, 22].

The only National Rabies Laboratory confirms every case of rabies in Ethiopia. The limited diagnostic facility, poor surveillance protocol, unavailability of vaccine and post-exposure treatment, an increasing stray dog population, low level of public awareness, poor attention and resource allocation by the government are major significant factors that hinder the control of rabies in Ethiopia [4, 17].

Scientific researches based on observational studies on viral isolation and identification is limited, except survey studies focused on knowledge assessment using questionnaire data. The true figure of the disease burden in the country remains unclear. Hence, this systematic review and meta-analysis summarize and pool estimates of the status of rabies in Ethiopia from previously published reports.

## Methods

The study was conducted based on the Preferred Reporting Items for Systematic reviews and Meta-Analyses (PRISMA) group checklist guideline [13]. The checklist was used to ensure the inclusion of relevant information (Additional file 1). The outcome of interest was the incidence of rabies in Ethiopia. Mendeley version 1.19.4 was used to catalog the initial literature search results.

### Literature search strategy and eligibility criteria

Literature was searched in PubMed, Science Direct and Google Scholar databases. A Boolean operator and/or was used during an online search by combining topic-related keywords. Key-words (MeSH terms) used when searching the databases were: rabies, dog bite, prevalence, incidence, and Ethiopia. The reference lists of all available studies on rabies in Ethiopia were checked to obtain additional literature. All searched articles were downloaded and titles and abstracts were assessed and respective papers were examined in detail.

### Inclusion/exclusion criteria

The inclusion criteria for studies include articles published in a reputable journal, written in English, and conducted in Ethiopia; studies conducted in animals or humans were considered inclusion criteria. Besides, cross-sectional, prospective cohort and retrospective studies regarding the incidence of dog bite exposure of rabies from 2010 to 2020 were our target studies.

### Data extraction procedure

Data were extracted using standardized Microsoft Excel sheet. Two independent researchers extracted data, and the disagreement was resolved by consensus based on the standardized extraction forms to guarantee consistency and accuracy. The following data were recorded in the extraction tables: paper identification (ID, first author, year of publication, title, journal, volume, page numbers), study type (cross-sectional, prospective cohort and Case-control studies), year of study, study locations were recorded).

### Statistical analysis

Pooled prevalence estimates were calculated using the random-effects model meta-analysis at 95% CI as substantial heterogeneity was expected [7]. Heterogeneity (I^2^) between studies was evaluated with Cochran’s Q test and inverse variance index. The I^2^ values of 25, 50, and 75% were considered low, moderate and high heterogeneity [10]. The I^2^ values, 0%, indicate no observed heterogeneity. Q is weighted of squares on a standardized scale. Low *p*-values were reported as the presence of heterogeneity [10].

Subgroup analysis was conducted according to regional distribution, study methods, sample size, and hosts (human and/ animals). Publication bias was assessed using the Begg and Egger’s test [8] and visual inspection of the funnel plot. Potentially contributing factors for between-study heterogeneity was evaluated using meta-regression. Multivariable meta regression analysis was done for variables like study location, affected host, study type and sample size. All statistical analyses were then computed using Stata version 13 software.

## Results

### Descriptive results

**Fig. 1:**
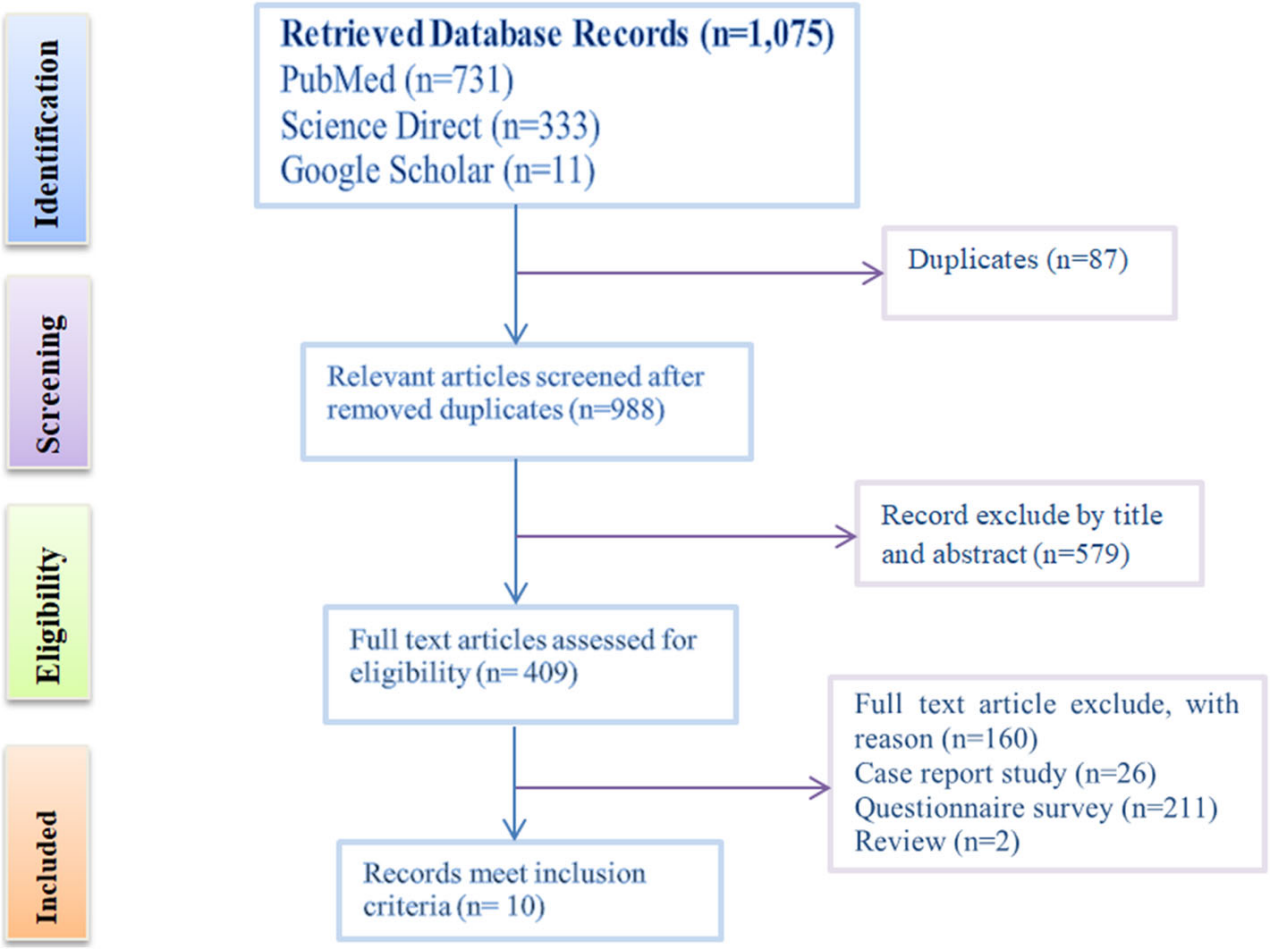
Flow diagram of the selection of eligible studies

From the total of 1075 studies retrieved, 10 articles with 20 data points met the inclusion criteria for the final meta-analysis (Fig. 1). The total sample size throughout the study years was 33,675 (2161 animals and 31,071 humans), of which 5249 were found positive for the rabies virus. The overall apparent prevalence from all studies was 32%. The largest study in terms of sample size employed 6874 humans, while the smallest had 278 humans. The disease was reported in five regions, including the Addis Ababa city administration. The virus was highly prevalent in Addis Ababa, Oromia and Tigray regional states. Detailed characteristics of the included studies in the meta-analysis are summarized in Table 1.

**Table 1:** Descriptive Metadata of rabies from 2010 to 2020 published studies in Ethiopia

Hosts affected	Study location	Study design	Sample size	No of positives	Prevalence	Reference
Humans	Amhara	Cross-sectional	532	83	15.6%	[11]
		Retrospective	3161	40	1.3%	[20]
		Retrospective	6527	423	6.5%	[20]
		Retrospective	6874	501	7.3%	[20]
		Retrospective	278	206	74.1%	[22]
		Retrospective	336	255	75.9%	[22]
	Nationwide	Retrospective	278	83	29.9%	[2]
		Retrospective	336	34	10.1%	[2]
		Prospective	3042	368	12.1%	[14]
		Retrospective	935	726	77.6%	[14]
	Oromia	Prospective	694	23	3.3%	[2]
	Tigray	Retrospective	832	747	89.8%	[18]
		Retrospective	861	630	73.2%	[18]
		Retrospective	3042	140	4.6%	[18]
		Retrospective	3101	81	2.6%	[18]
		Prospective	694	23	3.3%	[9]
Animals	Addis Ababa	Cross-sectional	532	83	15.6%	[17]
	Amhara	Retrospective	803	288	35.9%	[11]
	Oromia	Retrospective	817	515	63.0%	[12]
**Overall**			33,675	5249	32%	

### Meta-analysis

**Fig. 2:**
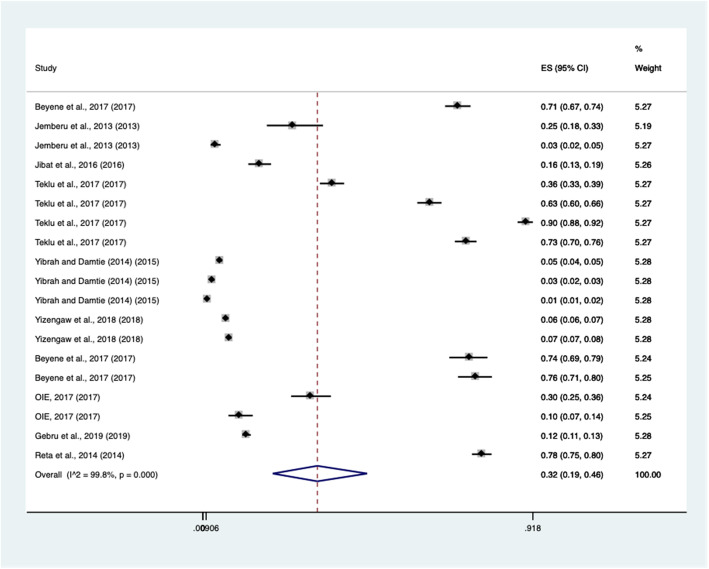
Forest plot for the pooled prevalence of rabies in Ethiopia from 2010 to 2020

The pooled prevalence of rabies in Ethiopia with a meta-analysis was estimated 32% (95% CI: 0.19–0.46). The individual study prevalence estimated estimates ranged from 1 to 78%. Studies were approximately weighted nearly equal with an individual weight ranging from 5.19–5.28%. The Forest plot for the pooled prevalence of rabies in Ethiopia is depicted in Fig. 2.

### Subgroup meta-analysis

. A subgroup meta-analysis was computed for host affected, location, study type and sample size because substantial heterogeneity was observed in the pooled estimate. Hence, sub-total random pooled prevalence of rabies was estimated at 28% (95% CI: 1–80%) in animals and 33% (95% CI: 0.20–0.47) in human (Table 2). Furthermore, subgroup analysis was computed for study location by clustering into regions. The highest prevalence was observed in Addis Ababa with a prevalence of 78% with 95% CI (75–80%), while the lowest prevalence was recorded in Amhara regional state with a prevalence of 5, 95% CI (3–8%).

**Table 2:** Subgroup Meta-statistics

Characteristics	No of observations	Pooled rabies prevalence				Heterogeneity	
		SS	Event	Event rate	95% CI	I^2^%	*P*
**Overall**	20	33,232	5640	0.32	0.19;0.46	99.85	0.001
**Region**						99.85	0.01
Addis Ababa	1	935	726	0.78	0.75;0.80	0	
Amhara	7	23,527	1240	0.05	0.03;0.08	98.3	
Nationwide	4	1228	578	0.47	0.14;0.80	99.4	
Oromia	2	1187	548	0.46	0.42;0.48	0	
Tigray	5	6355	2548	0.40	0.21;0.86	99.9	
**Host affected**							
Animal	3	2161	832	0.28	0.81;15.8	0	0.01
Human	17	31,071	4808	0.33	0.20;0.47	99.8	0.06
**Study type**							0.00
Prospective	3	3864	423	0.12	0.04;0.22	0	
Retrospective	15	27,845	4669	0.35	0.20;0.53	99.9	
Cross-sectional	2	1187	548	0.45	0.42;0.48	0	
**Sample size**							0.001
< 385	5	1356	610	0.45	0.21;0.65	99.3	
385–1000	8	6.129	3477	0.57	0.18;0.39	99.8	
> 1000	6	25,747	1553	0.06	0.02;0.08	99.4	

**Fig. 3:**
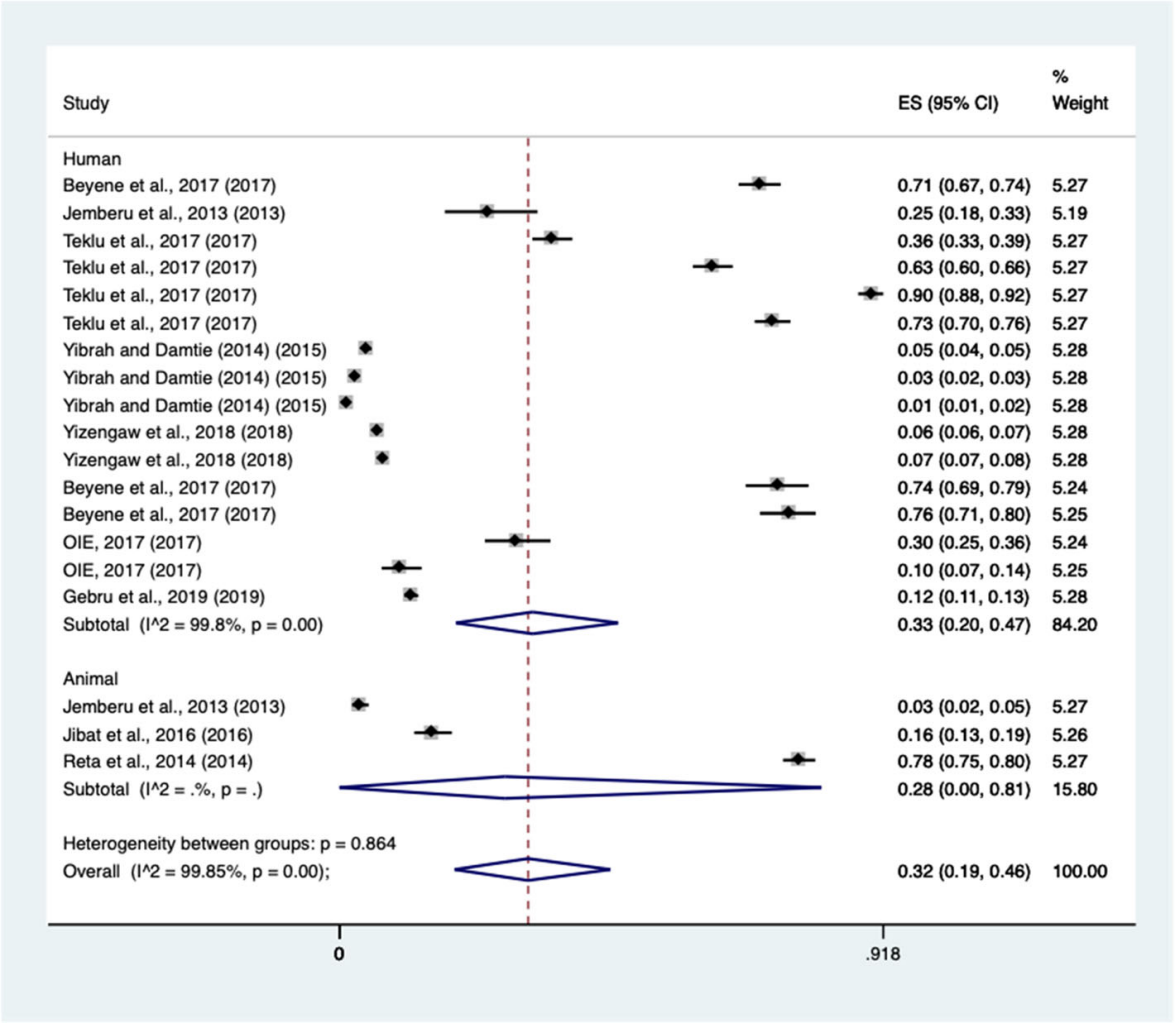
Subgroup meta-analysis by host species affected

**Fig. 4:**
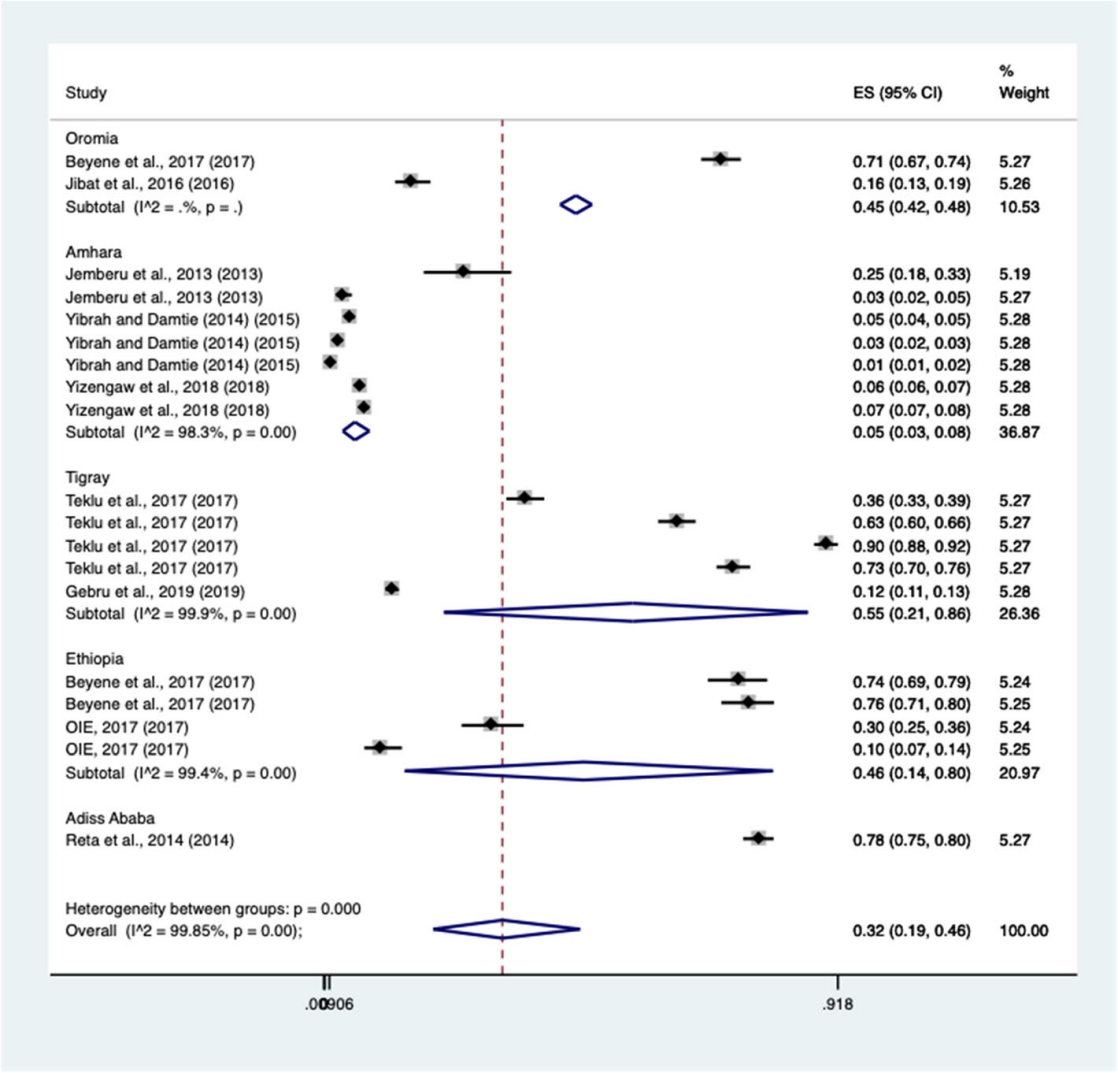
Subgroup meta-analysis by study regions

The subgroup analysis indicated that many studies were derived from the Amhara and Tigray regions. Study variability was observed in two locations and thus, results in such locations were omitted due to an insufficient number of observations. The subtotal pooled prevalence of rabies in the Amhara and Tigray regional state was 5% (CI: 0.03; 0.08%) and 55% (CI: 0.21; 0.86%), respectively. Despite this, one and two studies performed in Addis Ababa and Oromia regional state showed 78 and 45% pooled prevalence, respectively. The Forest plots of subgroup meta-analysis for host-affected and study regions are illustrated in Figs. 3 and 4.

### Meta-regression

Because the heterogeneity between studies remained after subgroup meta-analysis, meta regression was analyzed using variables like study region, study type, sample size and host affected were used in meta-regression. However, except for study regions and host affected which were marginally significant with a *p*-value of 0.06, none of the variables were significantly associated with event of rabies in the final multivariable meta-regression model (Table 3).

**Table 3:** Multivariable meta-regression analysis

Variables	Coefficient	***p***-value	95%CI
**Regions**			
**Addis Ababa**	*		
**Amhara**	−0.75	0.06	−1.35; − 0.14
**Oromia**	− 0.62	0.15	−1.47; − 0.23
**Tigray**	− 0.67	0.08	− 1.43; 0.08
**Nationwide**	−0.24	0.54	−1.03; 0.54
**Host**			
**Animal**	*		
**Human**	0.55	0.06	−0.04; 1.14
**Study type**			
**Prospective**	*		
**Retrospective**	−0.01	0.98	−0,44; 0.44
**Cross sectional**	**		
**Sample size**			
**< 385**	*		
**385–1000**	0.34	0.43	−0.50; 1.17
**> 1000**	−0.20	0.52	−0.81; 0.41

*Reference

** Omitted due to collinearity

### Publication bias and small study effect assessment

**Fig. 5:**
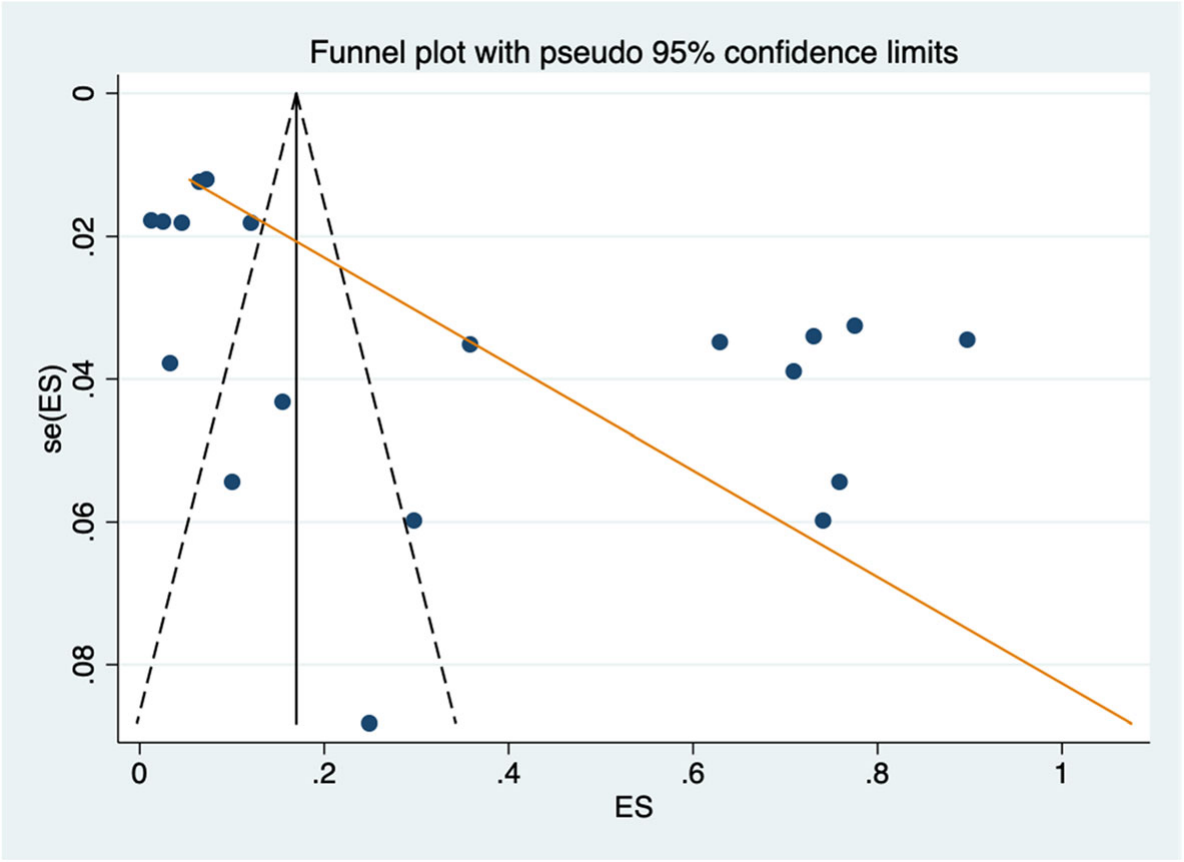
Funnel plot for assessing the presence of publication bias

The funnel plots (Fig. 5) and Egger’s regression asymmetry coefficient [b: 13.4] (CI: 5.43; 21.38: *p* < 0.05) suggest the presence of publication bias (Table 4).

**Table 4:** Egger’s test for small-study effect

	Standard effect	Coefficient	Std err	*P*-value	95% CI
Slope	− 0.11	0.09	− 1.19	0.250	−0.29; 0.08
Bias	13.41	3.78	3.55	0.002	5.43; 21.38

## Discussion

This is the first systematic review and meta-analyses on the incidence of rabies in Ethiopia to the best of our knowledge. The results presented in this report were from the analysis of data obtained through a systematic review of scientific publications of the prevalence of rabies at the country level between the years 2010 to 2020. Literature was heterogeneous, had inappropriate study designs, and unrepresentative sample size. This diversity, together with the lack of data on other required variables, reduced our dataset substantially. The final quantitative and meta-analysis of the prevalence were done only with 10 articles with 20 data points (records). Among them, 15 data points were on humans, three on animals and two records on both hosts. Relatively higher studies were in humans through a hospital-based retrospective analysis which is an indication that no active surveillances are undertaken in the country. On the other hand, even though the disease’s primary sources are animals, studies conducted on animals are very few. An increasing number of stray dogs in Ethiopia coupled with lack of dog vaccination practice and low public awareness creates difficulty controlling the disease. In the country, almost all animals are kept extensively exposing them to rabid dog bites. Most importantly absence of a post-exposure vaccine for animals made the condition worse.

The random effect meta-analysis result showed high variability with Higgin’s I^2^, which indicates that the variability between studies was not by chance alone. Because of the considerable variability between studies, the random effects meta-analysis weight of studies was nearly equal. The host affected and regions were marginally significant predictors. However, other variables in the final meta-regression remained statistically insignificant in explaining the study variability.

This review demonstrated that there is still a significantly higher prevalence of rabies in Ethiopia. A pooled prevalence of 32% at the country level needs critical attention from the responsible organizations. The pooled prevalence estimate varies significantly between regions which may be attributed to the number of reports. According to this systematic review and meta-analyses (SR&MA), rabies report has been mostly done in the Northern Region of Ethiopia, particularly in Amhara and Tigray regional states [11, 18, 20, 22]. Furthermore, higher number of reporting articles were implemented in human cases using retrospective data [2, 14, 17, 20]. Even though higher number of studies were reported from the Amhara regional state, the lowest prevalence was recorded in this region. However, the highest prevalence of rabies was reported from Addis Ababa. On the contrary, no study was reported from the country’s eastern and southern parts of Ethiopia to be included in this meta-analysis.

This review is a timely reminder of the need for more studies on animal reservoirs. Rabies has a devastating impact on poor third-world countries, with almost a hundred percent case fatalities in animals and humans [4, 12, 19]. Unlike Malaria, HIV/AIDS and TB, prioritization of rabies prevention is insufficient in Ethiopia; lack of focus on mass dog vaccination, uncontrolled animal movement and abounding free-roaming dogs makes the disease impact severe [1, 3, 4, 6, 16].

## Conclusions

To our knowledge, this is the first systemic review and meta-analysis attempt made on rabies in humans and animals. Our review revealed a high pooled prevalence of rabies and the disease had shown a significant variation among study regions. Relatively higher numbers of studies were done in Amhara and Tigray regional states. Therefore, more scientific researches need to be executed. Critical attention is needed from the country’s responsible bodies focusing on mass dog vaccination campaigns and public awareness to control the risk of rabies in the country.

## Supplementary Information

**Additional file 1.** PRISMA checklist

## Data Availability

All data presented on the main paper and raw datasets used and/or analyzed during the current study are available from the first author and corresponding author on reasonable request.

## Abbreviations

WHO: World Health Organization
PRISMA: Preferred Reporting Items for Systematic reviews and Meta-Analyses
EPHI: Ethiopian public health institute
MeSH: Medical Subject Heading

## References

[1] Ali A. National rabies survey preliminary report: household assessment. In: Proceedings of the National Workshop on Rabies Prevention and Control in Ethiopia. Adama; 2012. p. 81–90.

[2] Beyene TJ, Mourits MCM, Hogeveen H. Dog rabies data reported to multinational organizations from Southern and Eastern African countries. BMC Res Notes. 2017;10(1). 10.1186/s13104-017-2527-7. 10.1186/s13104-017-2527-7 PMC5465567 28595654

[3] Beyene TJ, Mourits MCM, Kidane AH, Hogeveen H (2018). Estimating the burden of rabies in Ethiopia by tracing dog bite victims. PLoS One.

[4] Coetzer A, Kidane AH, Bekele M, Hundera AD, Pieracci EG, Shiferaw ML, Wallace R, Nel LH (2016). The SARE tool for rabies control: current experience in Ethiopia. Antivir Res.

[5] Deressa A, Ali A, Bayene M, Selassie BN, Yimer E, Hussen K. The status of rabies in Ethiopia: a retrospective record review. Ethiop J Health Dev. 2010a;24(2). 10.4314/ejhd.v24i2.62961.

[6] Deribe K, Meribo K, Gebre T, Hailu A, Ali A, Aseffa A, Davey G (2012). The burden of neglected tropical diseases in Ethiopia, and opportunities for integrated control and elimination. Parasit Vectors.

[7] DerSimonian R, Laird N (1986). Meta-analysis in clinical trials. Control Clin Trials.

[8] Egger M, Davey Smith G, Schneider M, Minder C (1997). Bias in meta-analysis detected by a simple, graphical test. BMJ.

[9] Gebru G, Romha G, Asefa A, Hadush H, Biedemariam M. Risk factors and spatio-temporal patterns of human rabies exposure in Northwestern Tigray, Ethiopia. Ann Global Health. 2019;85(1):119. 10.5334/aogh.2518. 10.5334/aogh.2518 PMC6743033 31517464

[10] Higgins JPT, Thompson SG (2002). Quantifying heterogeneity in a meta-analysis.

[11] Jemberu WT, Molla W, Almaw G, Alemu S (2013). Incidence of rabies in humans and domestic animals and People’s awareness in North Gondar zone, Ethiopia. PLoS Negl Trop Dis.

[12] Jibat T, Mourits MCM, Hogeveen H (2016). Incidence and economic impact of rabies in the cattle population of Ethiopia. Prevent Vet Med.

[13] Moher D, Liberati A, Tetzlaff J, Altman DG, The PRISMA Group (2009). Preferred reporting items for systematic reviews and meta-analyses. PLoS Med.

[14] OIE (2017). Dog rabies data reported to multinational organizations from southern and eastern African countries. BMC Res Notes.

[15] Pankhurst R (1970). The history and traditional treatment of rabies in Ethiopia. Med Hist.

[16] Pieracci EG, Hall AJ, Gharpure R, Haile A, Walelign E, Deressa A (2016). Prioritizing zoonotic diseases in Ethiopia using a one health approach. One Health.

[17] Reta T, Teshale S, Deresa A, Ali A, Mengistu F, Sifer D, Freuling CM (2014). Rabies in animals and humans in and around Addis Ababa, the capital city of Ethiopia: A retrospective and questionnaire based study. J Vet Med.

[18] Teklu GG, Hailu TG, Eshetu GR (2017). High incidence of human rabies exposure in northwestern Tigray, Ethiopia: a four-year retrospective study. PLoS Negl Trop Dis.

[19] WHO. World Health Organization expert consultation on rabies. In: In Second report: Technical Report Series 982. Geneva; 2013. p. 8–67. 24069724

[20] Yibrah M, Damtie D. Incidence of human rabies exposure and associated factors at the Gondar health center, Ethiopia: a three-year retrospective study. Infect Dis Poverty. 2015;4(1). 10.1186/2049-9957-4-3. 10.1186/2049-9957-4-3 PMC4327962 25685347

[21] Yimer E, Newayeselassie B, Teferra G, Mekonnen Y, Bogale Y, Zewde B (2002). Situation of rabies in Ethiopia: a retrospective study 1990-2000. Ethiop J Health Dev.

[22] Yizengaw E, Getahun T, Mulu W, Ashagrie M, Abdela I, Geta M (2018). Incidence of human rabies virus exposure in northwestern Amhara, Ethiopa. BMC Infect Dis.

