# Supplementary material for: Prevalence of dog-mediated rabies in Ethiopia: a systematic review and Meta-analysis from 2010 to 2020
Source: One Health Outlook. 2021 Aug 4;3:16. doi: 10.1186/s42522-021-00046-7 (PMC8336362; doi:10.1186/s42522-021-00046-7)
Supplement: Supplementary file 1 — Additional file 1. PRISMA checklist [file 42522_2021_46_MOESM1_ESM.docx]

| Section/topic | # | Checklist item | Reported  on page # |
| --- | --- | --- | --- |
| TITLE | | |  |
| Title | 1 | **Status of dog- mediated Rabies in Ethiopia: Systemic review and Meta-analysis**. | 1 |
| ABSTRACT | | |  |
| Structured summary | 2 | Rabies is lethal zoonotic viral disease, mostly transmitted by dog-bite. Ethiopia accommodates the second largest number of rabies deaths of all African countries. The total number of animal rabies cases in Ethiopia is unknown. The objective of this systematic review and meta-analysis was to summarize and pool estimates the status of rabies in Ethiopia. Three bibliographic databases were comprehensively searched on PubMed indexed Journals through AND/OR Boolean operator between the years of 2010 to 2020. Eligible studies were selected following the removal of articles that failed to meet the inclusion criteria. The prevalence was estimated using the random effects meta-analysis and heterogeneity was evaluated by subgroup analysis. The pooled estimate of rabies across studies for the entire period was 32 % (95 % CI: 0.46; 100.00), with individual study prevalence estimates ranged from 1% to 78%. Studies were approximately equal with an individual weights ranging from 5.19% to 5.28%. Sub-total random pooled prevalence of rabies based up on hosts affected subgroup analysis was estimated at 28% (95% CI: 0.00; 0.81) in animals and 33% (95% CI: 0.20; 0.47) in human. There was a significant difference between study regions (p< 0.05). A considerable number of studies derived from Amhara and Tigray regional state was 5% (CI: 0.03; 0.08%) and 55% (CI: 0.21; 0.86%), respectively. Despite, subtotal-pooled prevalence of studies conducted on nationwide was found 46% (CI: 0.14; 0.80%). Nevertheless, one and two studies performed in Addis Ababa and Oromia regional state showed 78% and 45% pooled prevalence, respectively. No study was reported from eastern and southern parts of the country, this might be due to religious aspect that most of them are Muslim, cannot keep dogs in this region. Thus, the regional differences of data availability may have led to an over-estimation of the estimates for some regions. The estimated pooled rabies prevalence was found high and show varying among study regions. Therefore, Focusing on mass dog vaccination campaign along with public awareness should be implemented to control the disease. | 1 |
| INTRODUCTION | | |  |
| Rationale | 3 | Rabies has been reported as a significant public health threat in Ethiopia (12) and the control and elimination of rabies is a daunting undertaking and going as further challenged in Ethiopia. This is due to an increasing stray dog population, lack of rabies vaccines, poor rabies surveillance, low level of public awareness, poor attention and resource allocation by government are major important problems facing forward (6). Scientific researches based on observational studies on viral isolation and identification is limited except survey studies focused on knowledge assessment using questionnaire data. Furthermore, no data on systematic review and meta-analysis was done on rabies in Ethiopia. Hence, this systematic review and meta-analysis is conducted to summarize and pool estimates the status of rabies in Ethiopia and this help to indicate some basic practical measures for the government and policy makers for appropriate control strategies at national level. | 2 |
| Objectives | 4 | To summarize and pool estimates the status of rabies in Ethiopia and indicate some basic practical measures for the government and policy makers for appropriate control strategies at national level. | 2 |
| METHODS | | |  |
| Protocol and registration | 5 |  | NA |
| Eligibility criteria | 6 | Cross-sectional, prospective and retrospective (conducted for not more than five years) studies, regarding the incidence of dog bite exposure of rabies in Ethiopia during covering all dates in range of 2010 to 2020, published in a reputable journal, written in English, conducted in Ethiopia. | 2 |
| Information sources | 7 | PubMed, Science Direct and Google Scholar. All searched articles were downloaded and added to Mendeley reference manager. A Boolean operator AND/OR was used during online search through combining topic-related keywords. Key-words/MeSH terms | 2 |
| Search | 8 | PubMed=((("epidemiology"[MeSH Terms] OR "incidence "[MeSH Terms]) OR "prevalence "[MeSH Terms])) AND ("rabies"[MeSH Terms] AND ("ethiopia"[MeSH Terms])) | 2 |
| Study selection | 9 | Infected animals and/human, number of infected, study localities, size of study population and study method used were a criteria of selecting the study articles with excluding those survey and case studies. | 2 |
| Data collection process | 10 | Data were collected using electronic database of MEDLINE PubMed indexed journals with MeSH terms like rabies, dog bite, prevalence and Ethiopia. Data were extracted by two independent researchers | 2 |
| Data items | 11 | Paper identification (ID, first author, year of publication, title, journal, volume, page numbers), study design (cross-sectional, prospective cohort and case-control study), year of study, study Region). | 3 |
| Risk of bias in individual  studies | 12 | Describe methods used for assessing risk of bias of individual studies (including specification of whether this was done at the study or outcome level), and how this information is to be used in any data synthesis. | - |
| Summary measures | 13 | State the principal summary measures (e.g., risk ratio, difference in means). | - |
| Synthesis of results | 14 | Mean prevalence and standard error was initially calculated by considering numbers of events over total studied samples. Pooled prevalence estimates were calculated using the random-effects model meta-analysis at 95% CI as substantial heterogeneity was expected (17). Heterogeneity between studies was evaluated through the Cochran’s Q test and inverse variance index (I^2^), which describes the percentage of observed total variation between studies that are due to heterogeneity rather than chance. The I^2^ values of 25, 50, and 75% were considered low, moderate and high heterogeneity, respectively (18). The I^2^ values 0 % indicate no observed heterogeneity. Q is the weighted of squares on a standardized scale. Low p-values reported as the presence of heterogeneity (18). Subgroup analysis was conducted according to regional distribution, study methods, sample size and hosts affected (human and/ animals). Publication bias was assessed using the Begg and Egger’s test (19), and by visual inspection of the funnel plot (Fig. 2). Potentially contributing factors for between-study heterogeneity was evaluated using Meta-regression. Univariable and final multivariant analysis was done for variables like study location, affected host, study type and sample size. Statistical analyses were then computed using Stata software version 13 (Stata Corporation, College Station, USA). | 3 |
| Risk of bias across studies | 15 | Publication biases that affect the cumulative evidence since some studies wrere not published in journals indexed by PubMed and most of studies reported was questionnaire based survey and case studies which did not report the prevalence rate of infection. Therefore, we did not include these studies into our systemic review and Meta-analysis. | - |
| Additional analyses | 16 | subgroup analyses and meta-regression were performed. | 4 |
| RESULTS | | |  |
| Study selection | 17 | Ten articles with 20 different observations were selected in the final meta-analysis, which depicted in flow diagram. | 5-10 |
| Study characteristics | 18 | For each study, present characteristics for which data were extracted with its citation. |  |
| Risk of bias within studies | 19 | Present data on risk of bias of each study and, if available, any outcome level assessment (see item 12). |  |
| Results of individual studies | 20 | For all outcomes considered (benefits or harms), present, for each study: (a) simple summary data for each intervention group (b) effect estimates and confidence intervals, ideally with a forest plot. | 10 |
| Synthesis of results | 21 | Present results of each meta-analysis done, including confidence intervals and measures of consistency. | 10 |
| Risk of bias across studies | 22 | Present results of any assessment of risk of bias across studies (see Item 15). | - |
| Additional analysis | 23 | Give results of additional analyses, if done (e.g., sensitivity or subgroup analyses, meta-regression [see Item 16]). | 5-10 |
| DISCUSSION | | |  |
| Summary of evidence | 24 | Summarize the main findings including the strength of evidence for each main outcome; consider their relevance to  key groups (e.g., healthcare providers, users, and policy makers). | 11 |
| Limitations | 25 | Discuss limitations at study and outcome level (e.g., risk of bias), and at review-level (e.g., incomplete retrieval of  identified research, reporting bias). | 11 |
| Conclusions | 26 | Provide a general interpretation of the results in the context of other evidence, and implications for future research. | 11 |
| FUNDING | | |  |
| Funding | 27 | No fund | 12 |
